# Supplementary material for: MetaRibo-Seq measures translation in microbiomes
Source: Nat Commun. 2020 Jun 29;11:3268. doi: 10.1038/s41467-020-17081-z (PMC7324362; doi:10.1038/s41467-020-17081-z)
Supplement: Supplementary file 10 — Supplementary Data 7 [file 41467_2020_17081_MOESM10_ESM.zip › File2/Confidence_High_Taxonomy/369452_out.krona.html]

Javascript must be enabled to view this page.

members
magnitude
magnitudeUnassigned
count
unassigned
taxon
rank

369452\_out

22

2
superkingdom
22

976
phylum
22

200643
22
class

order
22
171549

22
family
815

816
genus

SRS017103\_contig\_number\_25713SRS017433\_contig\_number\_15562SRS018623\_contig\_number\_15009SRS023526\_contig\_number\_11379SRS056519\_contig\_number\_4991SRS075078\_contig\_number\_18513SRS075773\_contig\_number\_15596SRS144362\_contig\_number\_3451SRS145497\_contig\_number\_10368SRS147346\_contig\_number\_9855SRS893187\_contig\_number\_17199
22
11

species

SRS015431\_contig\_number\_76359SRS053398\_contig\_number\_15914
2
1946089

2292936

SRS098571\_contig\_number\_15372
1
species

species
1

SRS100021\_contig\_number\_2033
46506

species

SRS015782\_contig\_number\_39467SRS015960\_contig\_number\_2280SRS045713\_contig\_number\_15813SRS051610\_contig\_number\_8103SRS098717\_contig\_number\_14323SRS150029\_contig\_number\_12346
6
820

1

SRS147139\_contig\_number\_101
species
2292913
